# *catena*-Poly[(*trans*-diaqua­cadmium)-bis­{μ-5-[4-(1*H*-imidazol-1-yl)phen­yl]tetra­zol-1-ido}]

**DOI:** 10.1107/S1600536812014626

**Published:** 2012-04-13

**Authors:** Shao-Wei Tong, Shi-Jie Li, Wen-Dong Song, Dong-Liang Miao, Jing-Bo An

**Affiliations:** a College of Food Science and Technology, Guangdong Ocean University, Zhanjiang 524088, People’s Republic of China; b School of Enviroment Science and Engineering, Donghua University, Shanghai 200051, People’s Republic of China; c College of Science, Guangdong Ocean University, Zhanjiang 524088, People’s Republic of China

## Abstract

In the title compound, [Cd(C_10_H_7_N_6_)_2_(H_2_O)_2_], the Cd^II^ atom lies on an inversion centre and is coordinated by four N atoms from 5-[4-(1*H*-imidazol-1-yl)phen­yl]tetra­zol-1-ide ligands and two O atoms from the coordinated water mol­ecules in an octa­hedral arrangement. The complex polymeric chains are inter­connected *via* inter­molecular water O—H⋯N hydrogen bonds into a three-dimensional network.

## Related literature

For our previous work based on imidazole derivatives as ligands, see: Tong, Li *et al.* (2011 ▶); Li *et al.* (2010 ▶). For related structures, see: Huang *et al.* (2009 ▶); Cheng (2011 ▶). 
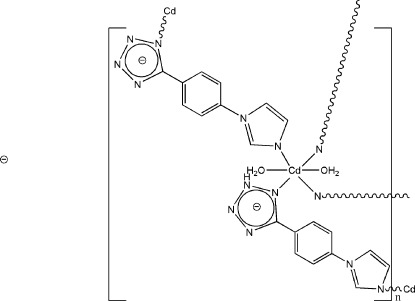


## Experimental

### 

#### Crystal data

[Cd(C_10_H_7_N_6_)_2_(H_2_O)_2_]
*M* *_r_* = 570.86
Triclinic, 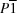

*a* = 7.6070 (6) Å
*b* = 8.0621 (8) Å
*c* = 9.1509 (9) Å
α = 102.762 (1)°
β = 97.495 (1)°
γ = 106.073 (2)°
*V* = 514.84 (8) Å^3^
*Z* = 1
Mo *K*α radiation
μ = 1.11 mm^−1^
*T* = 298 K
0.22 × 0.21 × 0.15 mm

#### Data collection

Bruker SMART 1000 CCD area-detector diffractometer
Absorption correction: multi-scan (*SADABS*; Bruker, 2007 ▶) *T*_min_ = 0.792, *T*_max_ = 0.851
2591 measured reflections
1768 independent reflections
1708 reflections with *I* > 2σ(*I*)
*R*_int_ = 0.015

#### Refinement

*R*[*F* ^2^ > 2σ(*F* ^2^)] = 0.027
*wR*(*F* ^2^) = 0.065
*S* = 1.14
1768 reflections
160 parameters
3 restraints
H-atom parameters constrained
Δρ_max_ = 0.48 e Å^−3^
Δρ_min_ = −0.62 e Å^−3^

### 

Data collection: *SMART* (Bruker, 2007 ▶); cell refinement: *SAINT* (Bruker, 2007 ▶); data reduction: *SAINT*; program(s) used to solve structure: *SHELXS97* (Sheldrick, 2008 ▶); program(s) used to refine structure: *SHELXL97* (Sheldrick, 2008 ▶); molecular graphics: *SHELXTL* (Sheldrick, 2008 ▶); software used to prepare material for publication: *SHELXTL*.

## Supplementary Material

Crystal structure: contains datablock(s) I, global. DOI: 10.1107/S1600536812014626/kp2399sup1.cif

Structure factors: contains datablock(s) I. DOI: 10.1107/S1600536812014626/kp2399Isup2.hkl

Additional supplementary materials:  crystallographic information; 3D view; checkCIF report

## Figures and Tables

**Table 1:** Selected bond lengths (Å)

Cd1—N6	2.264 (2)
Cd1—N1	2.385 (2)
Cd1—O1*W*	2.461 (2)

**Table 2:** Hydrogen-bond geometry (Å, °)

*D*—H⋯*A*	*D*—H	H⋯*A*	*D*⋯*A*	*D*—H⋯*A*
O1*W*—H1*W*⋯N4^i^	0.85	2.06	2.903 (3)	171
O1*W*—H2*W*⋯N3^ii^	0.85	2.11	2.953 (3)	171

Symmetry codes: (i) 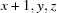; (ii) 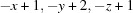.

**Fig. 1.**
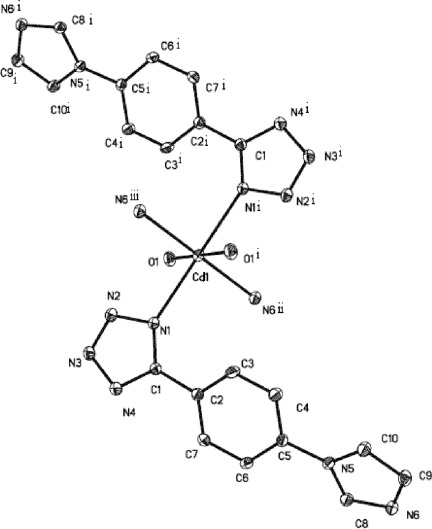
An asymmetric unit of (I)and atom numbering scheme for the title complex showing 30% probability ellipsoids. For symmetry codes: (i) -x + 3, -y + 1, -z + 1; (ii) -x + 2, -y, -z; (iii) x + 1, y + 1, z + 1.

**Fig. 2.**
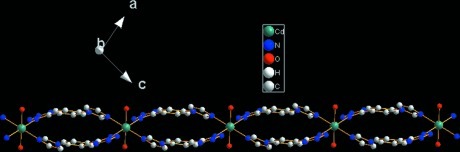
Polymeric chain of Cd(II) octahedra.

## supplementary crystallographic information

### Comment

The ligands having more N atoms can be used to synthesize complexes of variety of cordination modes. Our research group has show great interest in the metal-organic complexes with imidazole and tetrazole derivatives, such as 2-propyl-imidazole-4,5-dicarboxylic acid (Tong, Li *et al.*, 2011; Li *et al.*, 2010) and 1-tetrazole-4-imidazolebenzene. In this paper, we report the synthesis and structure of a new Cd^II^ complex, [Cd(C_8_H_9_N_2_O_4_)_4_(H_2_O)_2_]_n_ obtained under hydrothermal conditions. An asymmmetric unit of the title complex molecule includes one Cd^II^, 1-tetrazole-4-imidazolebenzene ligand and a coordinated water molecule (Fig. 1). The Cd^II^ atom is octahedrally coordinated and lies on an inversion centre, connected with four ligands [two imidazole N and two tetrazole N, Cd—N =2.264 (2) and 2.385 (2) Å] and two coordinated water molecules [Cd—O=2.461 (2) Å] (Table 1). The polymer chains (Fig. 2) are interconnected *via* water O—H···O and O—H···N hydrogen bonds (Table 2). For related structures of complexes with this ligand, see Huang *et al.* (2009) and Cheng (2011).

### Experimental

A mixture of cadmium nitrate (0.1 mmol, 0.020 g) and 1-tetrazole-4-imidazole-benzene (0.2 mmol, 0.043 g) in 12 mL of water and 3 mL of alcohol was sealed in an autoclave equipped with a Teflon liner (25 mL) and then heated at 413 K for 3 days. Crystals of the title compound were obtained by slow evaporation of the solvent at room temperature.

### Refinement

H atoms of the water molecule were located in a difference-Fourier map and refined as riding with an O—H distance restraint of 0.85 Å, with *U*_iso_(H) = 1.5 *U*_eq_. The imidazolyl and phenyl H atoms were located in a difference-Fourier but were refined as riding with C—H = 0.93 Å and *U*_iso_(H) = 1.5*U*_eq_(C).

### Crystal data

**Table:**

[Cd(C_10_H_7_N_6_)_2_(H_2_O)_2_]	*Z* = 1
*M**_r_* = 570.86	*F*(000) = 286
Triclinic, *P*1	*D*_x_ = 1.841 Mg m^−^^3^
Hall symbol: -P 1	Mo *K*α radiation, λ = 0.71073 Å
*a* = 7.6070 (6) Å	Cell parameters from 1702 reflections
*b* = 8.0621 (8) Å	θ = 2.5–25.9°
*c* = 9.1509 (9) Å	µ = 1.11 mm^−^^1^
α = 102.762 (1)°	*T* = 298 K
β = 97.495 (1)°	Block, colourless
γ = 106.073 (2)°	0.22 × 0.21 × 0.15 mm
*V* = 514.84 (8) Å^3^	

### Data collection

**Table:**

Bruker SMART 1000 CCD area-detector diffractometer	1768 independent reflections
Radiation source: fine-focus sealed tube	1708 reflections with *I* > 2σ(*I*)
Graphite monochromator	*R*_int_ = 0.015
φ and ω scans	θ_max_ = 25.0°, θ_min_ = 2.3°
Absorption correction: multi-scan (*SADABS*; Bruker, 2007)	*h* = −5→9
*T*_min_ = 0.792, *T*_max_ = 0.851	*k* = −9→8
2591 measured reflections	*l* = −10→8

### Refinement

**Table:**

Refinement on *F*^2^	Primary atom site location: structure-invariant direct methods
Least-squares matrix: full	Secondary atom site location: difference Fourier map
*R*[*F*^2^ > 2σ(*F*^2^)] = 0.027	Hydrogen site location: inferred from neighbouring sites
*wR*(*F*^2^) = 0.065	H-atom parameters constrained
*S* = 1.14	*w* = 1/[σ^2^(*F*_o_^2^) + (0.0345*P*)^2^ + 0.1705*P*] where *P* = (*F*_o_^2^ + 2*F*_c_^2^)/3
1768 reflections	(Δ/σ)_max_ < 0.001
160 parameters	Δρ_max_ = 0.48 e Å^−^^3^
3 restraints	Δρ_min_ = −0.62 e Å^−^^3^

### Special details

**Table:**

Geometry. All e.s.d.'s (except the e.s.d. in the dihedral angle between two l.s. planes) are estimated using the full covariance matrix. The cell e.s.d.'s are taken into account individually in the estimation of e.s.d.'s in distances, angles and torsion angles; correlations between e.s.d.'s in cell parameters are only used when they are defined by crystal symmetry. An approximate (isotropic) treatment of cell e.s.d.'s is used for estimating e.s.d.'s involving l.s. planes.
Refinement. Refinement of *F*^2^ against ALL reflections. The weighted *R*-factor *wR* and goodness of fit *S* are based on *F*^2^, conventional *R*-factors *R* are based on *F*, with *F* set to zero for negative *F*^2^. The threshold expression of *F*^2^ > σ(*F*^2^) is used only for calculating *R*-factors(gt) *etc*. and is not relevant to the choice of reflections for refinement. *R*-factors based on *F*^2^ are statistically about twice as large as those based on *F*, and *R*- factors based on ALL data will be even larger.

### Fractional atomic coordinates and isotropic or equivalent isotropic displacement parameters (Å^2^)

**Table:**

	*x*	*y*	*z*	*U*_iso_*/*U*_eq_	
Cd1	0.5000	0.5000	0.5000	0.02370 (13)	
N1	0.2660 (3)	0.6294 (3)	0.4304 (3)	0.0252 (6)	
N2	0.3282 (3)	0.8094 (3)	0.4926 (3)	0.0280 (6)	
N3	0.2042 (3)	0.8776 (3)	0.4406 (3)	0.0278 (6)	
N4	0.0567 (3)	0.7454 (3)	0.3421 (3)	0.0274 (6)	
N5	0.3041 (3)	0.1036 (3)	0.0476 (3)	0.0218 (5)	
N6	0.4348 (3)	0.3262 (3)	0.2564 (3)	0.0242 (5)	
O1W	0.6896 (3)	0.7364 (3)	0.4031 (3)	0.0297 (5)	
H2W	0.7079	0.8454	0.4492	0.045*	
H1W	0.7919	0.7268	0.3806	0.045*	
C1	0.0999 (4)	0.5951 (4)	0.3384 (3)	0.0215 (6)	
C2	−0.0149 (4)	0.4151 (4)	0.2423 (3)	0.0214 (6)	
C3	0.0003 (4)	0.2630 (4)	0.2830 (4)	0.0258 (7)	
H3	0.0763	0.2756	0.3757	0.031*	
C4	−0.0950 (4)	0.0934 (4)	0.1889 (3)	0.0259 (7)	
H4	−0.0818	−0.0071	0.2173	0.031*	
C5	−0.2105 (4)	0.0742 (4)	0.0518 (3)	0.0207 (6)	
C6	−0.2325 (4)	0.2233 (4)	0.0103 (4)	0.0284 (7)	
H6	−0.3123	0.2100	−0.0806	0.034*	
C7	−0.1346 (4)	0.3928 (4)	0.1053 (4)	0.0284 (7)	
H7	−0.1489	0.4931	0.0773	0.034*	
C8	0.3743 (4)	0.1495 (4)	0.2001 (3)	0.0241 (6)	
H8	0.3793	0.0683	0.2573	0.029*	
C9	0.4018 (4)	0.3952 (4)	0.1350 (4)	0.0272 (7)	
H9	0.4304	0.5167	0.1406	0.033*	
C10	0.3218 (4)	0.2606 (4)	0.0065 (4)	0.0272 (7)	
H10	0.2857	0.2717	−0.0910	0.033*	

### Atomic displacement parameters (Å^2^)

**Table:**

	*U*^11^	*U*^22^	*U*^33^	*U*^12^	*U*^13^	*U*^23^
Cd1	0.02649 (19)	0.02043 (18)	0.02061 (19)	0.00771 (13)	−0.00038 (12)	0.00108 (12)
N1	0.0254 (14)	0.0177 (12)	0.0279 (14)	0.0069 (10)	−0.0013 (11)	0.0009 (11)
N2	0.0273 (14)	0.0175 (12)	0.0335 (15)	0.0036 (11)	0.0016 (11)	0.0025 (11)
N3	0.0287 (14)	0.0188 (13)	0.0337 (15)	0.0072 (11)	0.0035 (11)	0.0043 (11)
N4	0.0273 (14)	0.0208 (13)	0.0311 (15)	0.0078 (11)	0.0007 (11)	0.0040 (11)
N5	0.0237 (13)	0.0185 (12)	0.0198 (13)	0.0049 (10)	0.0007 (10)	0.0026 (10)
N6	0.0262 (13)	0.0199 (12)	0.0237 (14)	0.0065 (10)	0.0030 (10)	0.0028 (10)
O1W	0.0283 (11)	0.0214 (11)	0.0388 (13)	0.0080 (9)	0.0079 (10)	0.0061 (10)
C1	0.0202 (14)	0.0209 (14)	0.0228 (16)	0.0075 (12)	0.0042 (12)	0.0039 (12)
C2	0.0183 (14)	0.0210 (14)	0.0234 (16)	0.0061 (11)	0.0045 (12)	0.0028 (12)
C3	0.0248 (16)	0.0256 (16)	0.0215 (16)	0.0034 (12)	−0.0035 (12)	0.0058 (13)
C4	0.0295 (16)	0.0213 (15)	0.0241 (16)	0.0037 (12)	0.0004 (13)	0.0084 (13)
C5	0.0216 (15)	0.0183 (14)	0.0203 (15)	0.0060 (11)	0.0038 (12)	0.0020 (12)
C6	0.0288 (17)	0.0259 (16)	0.0246 (17)	0.0085 (13)	−0.0067 (13)	0.0024 (13)
C7	0.0315 (17)	0.0214 (15)	0.0312 (18)	0.0124 (13)	−0.0035 (13)	0.0052 (13)
C8	0.0288 (16)	0.0217 (15)	0.0206 (16)	0.0072 (12)	0.0010 (12)	0.0066 (12)
C9	0.0359 (17)	0.0188 (15)	0.0265 (17)	0.0064 (13)	0.0050 (13)	0.0093 (13)
C10	0.0383 (18)	0.0202 (15)	0.0213 (16)	0.0067 (13)	0.0001 (13)	0.0087 (13)

### Geometric parameters (Å, º)

**Table:**

Cd1—N6	2.264 (2)	O1W—H1W	0.8500
Cd1—N6^i^	2.264 (2)	C1—C2	1.475 (4)
Cd1—N1	2.385 (2)	C2—C3	1.387 (4)
Cd1—N1^i^	2.385 (2)	C2—C7	1.395 (4)
Cd1—O1W^i^	2.461 (2)	C3—C4	1.380 (4)
Cd1—O1W	2.461 (2)	C3—H3	0.9300
N1—C1	1.345 (4)	C4—C5	1.387 (4)
N1—N2	1.356 (3)	C4—H4	0.9300
N2—N3	1.306 (4)	C5—C6	1.383 (4)
N3—N4	1.363 (3)	C5—N5^ii^	1.442 (3)
N4—C1	1.335 (4)	C6—C7	1.386 (4)
N5—C8	1.356 (4)	C6—H6	0.9300
N5—C10	1.375 (4)	C7—H7	0.9300
N5—C5^ii^	1.442 (3)	C8—H8	0.9300
N6—C8	1.326 (4)	C9—C10	1.347 (4)
N6—C9	1.373 (4)	C9—H9	0.9300
O1W—H2W	0.8500	C10—H10	0.9300
			
N6—Cd1—N6^i^	180.000 (1)	N4—C1—N1	111.2 (2)
N6—Cd1—N1	89.45 (8)	N4—C1—C2	125.0 (2)
N6^i^—Cd1—N1	90.55 (8)	N1—C1—C2	123.8 (2)
N6—Cd1—N1^i^	90.55 (8)	C3—C2—C7	118.3 (3)
N6^i^—Cd1—N1^i^	89.45 (8)	C3—C2—C1	120.5 (3)
N1—Cd1—N1^i^	180.000 (1)	C7—C2—C1	121.2 (3)
N6—Cd1—O1W^i^	94.50 (8)	C4—C3—C2	121.4 (3)
N6^i^—Cd1—O1W^i^	85.50 (8)	C4—C3—H3	119.3
N1—Cd1—O1W^i^	98.76 (8)	C2—C3—H3	119.3
N1^i^—Cd1—O1W^i^	81.24 (8)	C3—C4—C5	119.4 (3)
N6—Cd1—O1W	85.50 (8)	C3—C4—H4	120.3
N6^i^—Cd1—O1W	94.50 (8)	C5—C4—H4	120.3
N1—Cd1—O1W	81.24 (8)	C6—C5—C4	120.4 (3)
N1^i^—Cd1—O1W	98.76 (8)	C6—C5—N5^ii^	120.9 (3)
O1W^i^—Cd1—O1W	180.00 (7)	C4—C5—N5^ii^	118.7 (2)
C1—N1—N2	105.4 (2)	C5—C6—C7	119.5 (3)
C1—N1—Cd1	143.60 (19)	C5—C6—H6	120.3
N2—N1—Cd1	110.51 (17)	C7—C6—H6	120.3
N3—N2—N1	108.8 (2)	C6—C7—C2	120.9 (3)
N2—N3—N4	110.0 (2)	C6—C7—H7	119.5
C1—N4—N3	104.6 (2)	C2—C7—H7	119.5
C8—N5—C10	106.9 (2)	N6—C8—N5	110.7 (3)
C8—N5—C5^ii^	127.3 (2)	N6—C8—H8	124.7
C10—N5—C5^ii^	125.5 (2)	N5—C8—H8	124.7
C8—N6—C9	106.0 (2)	C10—C9—N6	109.8 (3)
C8—N6—Cd1	131.1 (2)	C10—C9—H9	125.1
C9—N6—Cd1	120.68 (19)	N6—C9—H9	125.1
Cd1—O1W—H2W	118.8	C9—C10—N5	106.6 (3)
Cd1—O1W—H1W	117.9	C9—C10—H10	126.7
H2W—O1W—H1W	108.2	N5—C10—H10	126.7
			
N6—Cd1—N1—C1	32.7 (4)	Cd1—N1—C1—N4	−170.3 (2)
N6^i^—Cd1—N1—C1	−147.3 (4)	N2—N1—C1—C2	177.5 (3)
N1^i^—Cd1—N1—C1	139 (100)	Cd1—N1—C1—C2	7.6 (5)
O1W^i^—Cd1—N1—C1	−61.8 (4)	N4—C1—C2—C3	−156.3 (3)
O1W—Cd1—N1—C1	118.2 (4)	N1—C1—C2—C3	26.0 (4)
N6—Cd1—N1—N2	−136.9 (2)	N4—C1—C2—C7	26.6 (5)
N6^i^—Cd1—N1—N2	43.1 (2)	N1—C1—C2—C7	−151.0 (3)
N1^i^—Cd1—N1—N2	−30 (100)	C7—C2—C3—C4	2.2 (5)
O1W^i^—Cd1—N1—N2	128.65 (19)	C1—C2—C3—C4	−175.0 (3)
O1W—Cd1—N1—N2	−51.35 (19)	C2—C3—C4—C5	−0.9 (5)
C1—N1—N2—N3	0.4 (3)	C3—C4—C5—C6	−0.9 (5)
Cd1—N1—N2—N3	174.02 (19)	C3—C4—C5—N5^ii^	177.9 (3)
N1—N2—N3—N4	−0.2 (3)	C4—C5—C6—C7	1.5 (5)
N2—N3—N4—C1	−0.1 (3)	N5^ii^—C5—C6—C7	−177.3 (3)
N6^i^—Cd1—N6—C8	−60 (100)	C5—C6—C7—C2	−0.3 (5)
N1—Cd1—N6—C8	−119.3 (3)	C3—C2—C7—C6	−1.5 (5)
N1^i^—Cd1—N6—C8	60.7 (3)	C1—C2—C7—C6	175.6 (3)
O1W^i^—Cd1—N6—C8	−20.6 (3)	C9—N6—C8—N5	0.0 (3)
O1W—Cd1—N6—C8	159.4 (3)	Cd1—N6—C8—N5	162.55 (19)
N6^i^—Cd1—N6—C9	101 (100)	C10—N5—C8—N6	0.0 (3)
N1—Cd1—N6—C9	41.1 (2)	C5^ii^—N5—C8—N6	−174.1 (2)
N1^i^—Cd1—N6—C9	−138.9 (2)	C8—N6—C9—C10	0.0 (3)
O1W^i^—Cd1—N6—C9	139.9 (2)	Cd1—N6—C9—C10	−164.8 (2)
O1W—Cd1—N6—C9	−40.1 (2)	N6—C9—C10—N5	0.0 (4)
N3—N4—C1—N1	0.3 (3)	C8—N5—C10—C9	0.0 (3)
N3—N4—C1—C2	−177.6 (3)	C5^ii^—N5—C10—C9	174.3 (3)
N2—N1—C1—N4	−0.5 (3)		

Symmetry codes: (i) −*x*+1, −*y*+1, −*z*+1; (ii) −*x*, −*y*, −*z*.

### Hydrogen-bond geometry (Å, º)

**Table:**

*D*—H···*A*	*D*—H	H···*A*	*D*···*A*	*D*—H···*A*
O1*W*—H1*W*···N4^iii^	0.85	2.06	2.903 (3)	171
O1*W*—H2*W*···N3^iv^	0.85	2.11	2.953 (3)	171

Symmetry codes: (iii) *x*+1, *y*, *z*; (iv) −*x*+1, −*y*+2, −*z*+1.
